# Determinants of Vitamin D Levels in Children and Adolescents with Down Syndrome

**DOI:** 10.1155/2015/896758

**Published:** 2015-01-20

**Authors:** Stefano Stagi, Elisabetta Lapi, Silvia Romano, Sara Bargiacchi, Alice Brambilla, Sabrina Giglio, Salvatore Seminara, Maurizio de Martino

**Affiliations:** ^1^Health Sciences Department, Anna Meyer Children's University Hospital, University of Florence, Viale Pieraccini 24, 50139 Florence, Italy; ^2^Genetics and Molecular Medicine Unit, Anna Meyer Children's University Hospital, Viale Pieraccini 24, 50139 Florence, Italy

## Abstract

*Background*. Poor studies have evaluated 25-hydroxycholecalciferol (25(OH)D) levels in Down syndrome (DS).* Objective*. To assess in DS subjects serum 25(OH)D value, to identify risk factors for vitamin D deficiency, and to evaluate whether a normal 25(OH)D value can be restored with a 400 I.U. daily supplement of cholecalciferol in respect to controls.* Methods*. We have longitudinally evaluated 31 DS patients (aged 4.5–18.9 years old) and 99 age- and sex-matched healthy controls. In these subjects, we analysed calcium, phosphate, parathyroid hormone (PTH), 25(OH)D concentrations, and calcium and 25(OH)D dietary intakes, and we quantified outdoor exposure. After 12.3 months (range 8.1–14.7 months) of 25(OH)D supplementation, we reevaluated these subjects.* Results*. DS subjects showed reduced 25(OH)D levels compared to controls (*P* < 0.0001), in particular DS subjects with obesity (*P* < 0.05) and autoimmune diseases history (*P* < 0.005). PTH levels were significantly higher in DS subjects than controls (*P* < 0.0001). After cholecalciferol supplementation, 25(OH)D levels were significantly ameliorated (*P* < 0.05), even if reduced compared to controls (*P* < 0.0001), in particular in DS subjects with obesity (*P* < 0.05) and autoimmune diseases (*P* < 0.001).* Conclusions*. Hypovitaminosis D is very frequent in DS subjects, in particular in presence of obesity and autoimmune diseases. In these subjects, there could be a need for higher cholecalciferol supplementation.

## 1. Introduction

Down syndrome (DS) is the most common genetic (chromosomal) mental retardation syndrome, occurring in 1 in 700–1000 live births [1]. In DS, common features include distinctive craniofacial features, congenital heart disease, middle ear disease, and immune and endocrine system abnormalities [2, 3].

In recent years, we have witnessed a growing interest in the mass and bone quality of patients with DS; many studies have evaluated densitometric characteristics via dual-energy X-ray absorptiometry (DXA) [4–7], peripheral quantitative computed tomography (pQCT) [8], and quantitative ultrasound (QUS) [9]. However, many studies have focused on adults who live either in the community or in residential institutions [10]. In these patients, several environmental and hormonal factors such as muscle hypotonia, low physical activity, poor calcium and vitamin D intake, hypogonadism, growth retardation, and thyroid dysfunction may contribute to low bone mineral density (BMD) [4, 9]. These patients may develop reduced bone-mass accrual, predisposing them to fragility, fractures, and osteoporosis.

Among these factors, vitamin D may play a significant role in the health of patients with DS. Vitamin D status varies widely between different countries in Europe [11, 12], depending on many factors such as different exposures to sunshine, dietary intake of vitamin D, and the use of supplements [13].

Normally, in winter months and with increasing latitude, the amount of ultraviolet radiation reaching the earth's atmosphere is decreased, because the rays of the sun enter the atmosphere at a more oblique angle. As a consequence, little vitamin D is produced in the skin [11]. Moreover, during the winter months, children spend more time indoors and less of their skin is exposed to the sun. This fact may explain the high percentage of DS individuals and controls showing a vitamin D deficiency.

Vitamin D status is defined according to the serum concentration of 25-hydroxyvitamin D (25(OH)D). As previously reported, vitamin D deficiency is defined to exist when the serum 25(OH)D level is lower than 25 nmol/L (10 ng/mL); vitamin D insufficiency is considered to exist when the serum 25(OH)D is between 25 and 50 nmol/L (10–20 ng/mL) [14, 15]. However, an evaluation of satisfactory levels of vitamin D in healthy children has not yet been reported by adequate studies [15].

The prevalence of 25(OH)D deficiency varies between 30% and 93% in different studies in adults [16], even if in Norway and Sweden the prevalence is rather low [17]. However, in a small Italian study [18], more than 80% of children showed insufficiency or deficiency of 25(OH)D; the data were confirmed in other, larger studies providing cross-sectional [19] and longitudinal data [20].

An adequate status of 25(OH)D is very important because 25(OH)D deficiency has been shown to be a risk factor for several chronic diseases, in addition to the classic deleterious effects on bone, such as secondary hyperparathyroidism, and reduced bone accrual and mass [21]. In fact, the discovery that most tissues in the body have vitamin D receptors and several have one hydroxylase enzyme to convert 25(OH)D to its active form has provided new insights into the pleiotropic role of this vitamin [21].

Emerging evidence suggests that vitamin D also plays an important role in immune regulation. In fact, vitamin D receptors are found on several immune cells and vitamin D metabolites seem to modulate T cell proliferation and dendritic cell function [21, 22]. However, vitamin D deficiency may be a risk factor for the development of autoimmune diseases [22] and loss of muscle mass and muscle weakness [23]. Finally, many data have demonstrated that vitamin D may confer protection against diabetes mellitus (DM) type 1, hypertension, multiple sclerosis, and cancer [24, 25].

Therefore, vitamin D insufficiency may have important health consequences because of its role in the maintenance of normal bone mass turnover and its role as an immunoregulatory agent.

To date, few studies have assessed vitamin D status among children and adults with DS [26, 27]. Such studies have yielded conflicting results about the beneficial effects of intervention with vitamin D [26] or the lack of prescribing vitamin D when appropriate periods of exposure to sunlight exposure are available [27].

The purpose of the present study is to assess serum 25(OH)D in children and adolescents living in Tuscany, Italy, (latitude: 44° north), and to identify risk factors for vitamin D deficiency in different age groups of individuals with DS. Furthermore, this study also aimed to evaluate whether a normal 25(OH)D value can be restored in 25(OH)D-deficient DS patients in respect to controls with a daily supplement of 400 I.U. of cholecalciferol and by improving the factors influencing 25(OH)D status.

## 2. Methods

We longitudinally evaluated 31 Caucasian children and adolescents (17 males and 14 females, aged 4.5–18.9 years) with DS from Tuscany in the central region of Italy. All of the subjects were selected among individuals with DS who visited the Paediatric Endocrinology Unit of Anna Meyer Children's University Hospital in Florence and the Paediatric Unit, Mugello's Hospital, Borgo San Lorenzo, Italy, between December 2010 and October 2013.

The Hospital Ethics Committees of Anna Meyer Children's University Hospital and Mugello's Hospital approved the study, conducted in accordance with the Declaration of Helsinki guidelines. All of the subjects and/or their guardians signed documents of informed consent.

### 2.1. Study Design

The present study was a 12-month (*T*_0_-*T*_1_) controlled study of vitamin D supplementation. For baseline data collection, the DS patients were compared with a 1 : 3 proportion of healthy Caucasian controls.

Of the 38 DS subjects initially recruited to take part in this interventional study, 7 dropped out for various reasons (noncompliance, lost to follow-up, etc.). The final count, 31 individuals (81.6%), was the subjects who were able to finish the interventional study.

At *T*_0_ and *T*_1_, clinical and demographic data were collected from both the DS patients and healthy controls, including height, weight, body mass index (BMI), blood pressure, pubertal stage, therapies carried out, family and patient histories of autoimmune diseases and osteoporosis, and time dedicated to outdoor physical activity. Furthermore, nutrients diaries were recorded for each patient based on medical charts and standardized interviews.

However, during the longitudinal study, the DS subjects and healthy controls were divided into two age groups: children (2–12 years) and adolescents (older than 12 years).

At *T*_0_ and *T*_1_, all of the DS patients and healthy controls underwent laboratory tests to measure their plasma 25(OH)D levels, serum calcium and phosphate, bone specific alkaline phosphatase, parathyroid hormone (PTH), triglycerides (TG), total cholesterol, and low-density lipoprotein (LDL) cholesterol.

None of the participants had a recent history of travelling to warmer, sunnier areas prior to and/or during the study. Other exclusion criteria included taking calcium, vitamin D supplements, or any drugs affecting calcium or vitamin D metabolism in the past six months, such as a positive history of primary hyperparathyroidism or other skeletal diseases, severe obesity, malabsorptive disorders, and neurological or renal diseases.

Vitamin D status: serum 25-OH levels were stratified according to the following brackets: ≤10, 11–20, and 21–30 and >30 ng/mL, and they were defined as severe deficiency, deficiency, insufficiency, and sufficiency, respectively, according to previously established guidelines for bone health (in the absence of a consensus regarding appropriate levels for endocrine and extraendocrine health) [28, 29].

However, for evaluating the seasonal variations of 25(OH)D, we divided the year into four seasons: winter (December–March), spring (March–June), summer (June–September), and autumn (September–December).

Evaluation of dietary intake of calcium and vitamin D: dietary intakes of calcium and 25(OH)D were estimated using standardized interviews (by the parents) recording race, religion, country of birth, birth weight, type of feeding during the first year (breast, formula milk, or mixed), mother's use of vitamin supplementation during her pregnancy, child's use of vitamin D supplementation, and daily intake of cow's milk (categorized as more or less than 200 mL per day) [30].

Nutrient analyses were obtained from the Food Composition Database for Epidemiological Studies in Italy [31]. The frequency consumption (daily, weekly, and monthly) of each food item was evaluated.

Outdoor exposure evaluation: outdoor exposure was quantified from both questions regarding each child's and adolescent's average number of daily outdoor hours across each season and a prospective daily time-activity diary. For this analysis, we used an activity questionnaire and physical activity was assessed with a modified activity score composed of the scores for outdoor sports/leisure activities (0, <2, or >2 hours per week), as previously described [32].

Vitamin D intervention: all of the subjects (DS patients and controls) were treated with 400 I.U. (10 *μ*g) of vitamin D3 (cholecalciferol) administered orally once daily from November through May. The vitamin D3 supplement was purchased from Abiogen Pharma S.p.A. (Pisa, Italy), and the drops contained 250 I.U. of vitamin D3. The administration was based on the recommendation of the American Academy of Pediatrics that stated a recommended daily intake of vitamin D of 400 I.U./day for all infants, children, and adolescents [33, 34].

After 25(OH)D supplementation, 25(OH)D levels, serum calcium and phosphate, alkaline phosphatase, and PTH were reevaluated. The mean elapsed duration between the first and the second determinations was 12.3 months (range: 8.1–14.7 months).

Compliance was evaluated by written instructions given at the onset of the study and at clinical controls through the delivery of a written questionnaire drawn up by the parents. Compliance was further verified by e-mails and/or telephone interviews performed by a study nurse (to confirm the 25(OH)D intake) and by the bottle count performed at the end of the study period.

### 2.2. Control Group

Control group included 99 (84.6% out of 117 recruited subjects initially) healthy age- and sex-matched subjects (51 males and 48 females: age range 4.8–19.8 years) seen for noninflammatory musculoskeletal complaints. All of the subjects were evaluated at the time of routine follow-up visits, and parental informed consent was obtained.

### 2.3. Methods

Height was measured using Harpenden's stadiometer in triplicate to the nearest 0.1 cm. Weight was determined to the nearest 0.1 kg using a balance scale. BMI was calculated using the formula BMI = weight (kg)/height (m^2^). DS age-related reference values for height and BMI were used [35]. However, age-related reference values for height and BMI currently used in Italy, obtained in high sample numbers of Italian children, were used for comparison between DS subjects and controls [36]. Subjects with a BMI over the 95th percentile were considered obese, and subjects with a BMI over the 85th percentile but below the 95th percentile were considered overweight [37].

As described, height and BMI were normalized for chronological age by converting to standard deviation scores (SDSs). SDSs were calculated according to the following formula: patient value minus mean of age-related reference value/standard deviation of the age-related reference value [38].

Pubertal staging was determined at baseline and at each visit and was performed according to the criteria of Marshall and Tanner [39, 40], using an orchidometer in boys.

Blood pressure was measured three times by trained personnel by auscultation using a mercury sphygmomanometer on the right arm after the patient has been sitting quietly for 5 minutes, with the back supported, feet on the floor, right arm supported, and cubital fossa at heart level, as previously described [41].

Blood samples were obtained from each study participant after an overnight fast. Plasma concentrations of calcium, phosphate, and alkaline phosphatase were determined following routine biochemical laboratory protocols. Furthermore, the total cholesterol and triglyceride (TG) measurements were performed according to routine laboratory methods. Low-density lipoprotein (LDL) cholesterol was calculated using the Friedwald formula: LDL = total cholesterol − HDL cholesterol − TG/2.2.

Sera 25(OH)D and PTH were determined by chemiluminescence enzyme-labeled immunometric assays using an IMMULITE 2000 Systems analyzer (Siemens, Gwynedd, UK). The intra- and interassays CVs were <5% and <8% and <8% and <10%, respectively.

### 2.4. Statistical Analyses

Statistical analyses were performed using SPSSX (SPSSX Inc., Chicago, IL, USA). Clinical variables considered relevant to the study were as follows: sex (M : F), BMI SDSs, height SDSs, age at onset of puberty, pubertal stage, plasma concentrations of calcium, phosphate, alkaline phosphatase, and sera 25(OH)D and PTH at the first and second examinations. The characteristics of the study population were described through frequency distributions for categorical variables and through means and standard deviations (SDs), medians, and range for continuous variables.

For categorical variables, we used the *χ* ^2^ test and Fisher's exact test. The Kolmogorov-Smirnov test was used to determine if variables were normally distributed. For continuous variables, groups were compared using Student's *t*-test and Mann-Whitney *U* test, since not all of the continuous variables were normally distributed according to Shapiro-Wilk's test. Intergroup comparisons for parameters were conducted using analysis of variance (ANOVA) or repeated-measures analysis of covariance (ANCOVA), as appropriate. Spearman's and/or Pearson's correlation test was used to determine correlation coefficients. A multiple stepwise regression was performed to investigate factors associated with insufficient vitamin D status, after adjusting for potential confounders (age, sex, pubertal stage, vitamin D intake, and BMI). Covariates that were found to be nonsignificant at the 0.05 level were removed from the regression model using a stepwise elimination technique. All *P* values <0.05 were considered to be statistically significant.

## 3. Results

The baseline and longitudinal data of the study are reported in Table 1.

### 3.1. Baseline Data

No statistically significant differences in terms of history of fractures were found between our group of patients with DS and the control group. On the contrary, a statistically significant difference was found regarding height SDSs (−1.5 ± 1.0 versus −0.2 ± 0.8; *P* < 0.005) and the BMI SDSs (1.0 ± 1.4 versus −0.1 ± 0.9; *P* < 0.05), even when considering the children and adolescents separately. Furthermore, no statistical differences were found regarding 25(OH)D status in the different seasons between DS patients and controls.

### 3.2. Baseline 25(OH)D Level

Evaluating the percentages regarding 25(OH)D sufficiency, insufficiency, and deficiency, 2/31 (6.5%) of DS subjects had sufficient vitamin D levels, 5/31 (16.1%) had insufficient levels, 14/31 (45.2%) showed deficient levels, and 10/31 (32.2%) exhibited a severe deficiency. The percentage of 25(OH)D sufficiency is not significantly different from the controls (11/99: 11.1%), but the insufficiency (33/99: 33.3%; *P* < 0.005), deficiency (35/99: 35.4%; *P* < 0.05), and severe deficiency (20/99: 20.2%; *P* < 0.005) are significantly different.

**Figure 1:**
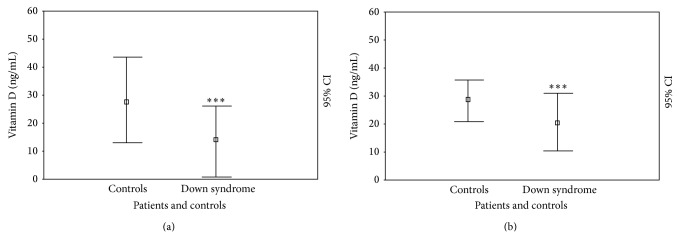
25(OH)D levels (ng/mL) at cross-sectional (a) and longitudinal (b) evaluation in patients with Down syndrome and controls. ^*^ *P* < 0.05; ^**^ *P* < 0.005; ^***^ *P* < 0.001.

**Figure 2:**
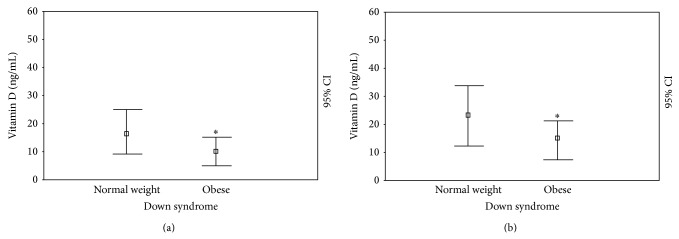
25(OH)D levels (ng/mL) at cross-sectional (a) and longitudinal (b) evaluation in patients with Down syndrome and obesity and normal weight. ^*^ *P* < 0.05; ^**^ *P* < 0.005; ^***^ *P* < 0.001.

**Figure 3:**
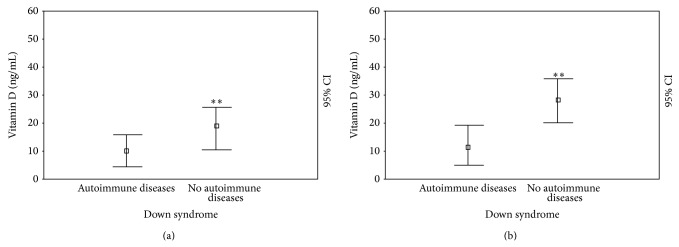
25(OH)D levels (ng/mL) at cross-sectional (a) and longitudinal (b) evaluation in patients with Down syndrome with and without autoimmune diseases. ^*^ *P* < 0.05; ^**^ *P* < 0.005; ^***^ *P* < 0.001.

However, for evaluating the 25(OH)D levels, we show that the DS subjects had significantly reduced 25(OH)D levels compared with the controls (14.34 ± 8.31 ng/mL versus 27.04 ± 7.47; *P* < 0.0001) (Figure 1(a)). In the DS subjects, 25(OH)D levels were not different between males (14.85 ± 8.25 ng/mL) and females (13.75 ± 8.70), children (14.26 ± 8.71 ng/mL), and adolescents (14.45 ± 8.15); we showed significant statistical differences between DS individuals with normal weights (16.93 ± 8.71 ng/mL) and those who were obese (10.20 ± 5.13; *P* < 0.05) (Figure 2(a)) and DS individuals without (19.00 ± 8.06 ng/mL) and with (10.35 ± 6.57 ng/mL; *P* < 0.005) a history of autoimmune diseases (Figure 3(a)).

**Figure 4:**
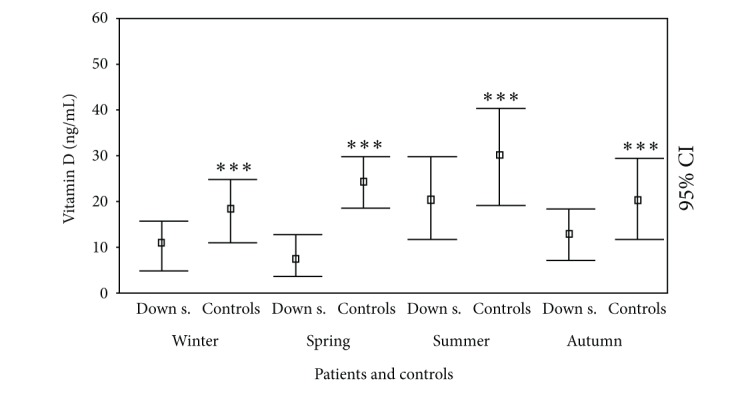
25(OH)D levels (ng/mL) at cross-sectional evaluation in patients with Down syndrome and controls in different seasons. ^*^ *P* < 0.05; ^**^ *P* < 0.005; ^***^ *P* < 0.001.

Regarding the effect of the different seasons on 25(OH)D status, the levels of 25(OH)D in DS subjects were significantly reduced in winter, spring, and autumn (11.33 ± 5.16, 7.85 ± 4.67, and 12.42 ± 4.96 ng/mL, resp.) with respect to summer value (22.53 ± 8.87 ng/mL; *P* < 0.005, *P* < 0.001, and *P* < 0.05, resp.) (Figure 4). These results were not significantly different when DS patients were divided into a child group (winter: 12.46 ± 5.67; spring: 8.98 ± 5.02; summer: 23.12 ± 8.76; autumn: 13.43 ± 5.22 ng/mL) and an adolescent group (winter: 10.12 ± 4.89; spring: 7.34 ± 4.65; summer: 21.00 ± 8.99; autumn: 11.76 ± 4.54 ng/mL) and males (winter: 11.88 ± 5.34; spring: 8.13 ± 4.88; summer: 23.65 ± 7.80; autumn: 11.37 ± 4.34 ng/mL) and females (winter: 10.89 ± 5.00; spring: 7.67 ± 4.34; summer: 22.02 ± 8.99; autumn: 13.13 ± 4.99 ng/mL).

However, these values are always significantly smaller in DS individuals than in controls (winter: 18.9 ± 6.3 ng/mL; *P* < 0.005; spring: 24.82 ± 6.06 ng/mL; *P* < 0.0001; summer: 30.33 ± 11.03 ng/mL; *P* < 0.005; autumn: 21.23 ± 6.24 ng/mL; *P* < 0.005) (Figure 4).

### 3.3. Baseline Calcium-Phosphate and PTH Level

**Figure 5:**
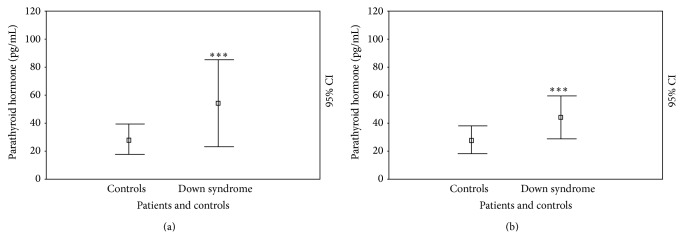
Parathyroid hormone levels (ng/mL) at cross-sectional (a) and longitudinal (b) evaluation in patients with Down syndrome and controls. ^*^ *P* < 0.05; ^**^ *P* < 0.005; ^***^ *P* < 0.001.

DS patients showed normal total calcium levels (2.42 ± 0.14 versus 2.51 ± 0.10 mmol/L; *P* = NS) compared with the controls and their phosphate levels were normal. However, DS patients also had significantly higher PTH levels compared with the controls (54.76 ± 32.15 versus 26.13 ± 10.76 pg/mL; *P* < 0.0001) (Figure 5(a)). PTH levels were higher, but not significantly, in DS adolescents (60.50 ± 38.45 pg/mL) compared with children (46.30 ± 27.93 pg/mL). These results differ from what is seen in the controls, in which we showed that PTH levels in children were 25.23 ± 9.10 pg/mL and 26.87 ± 8.57 pg/mL in adolescents; a statistical difference between DS individuals and controls was seen in children (*P* < 0.0001) but not in adolescents.

### 3.4. Baseline Dietary Evaluation

No significant differences were found in calcium intake between DS patients and the controls (796 ± 283 versus 821 ± 256 mg/day), even if the samples were divided into child (810 ± 270 versus 835 ± 285 mg/day) and adolescent (755 ± 290 versus 790 ± 235 mg/day) groups. Nevertheless, despite the dietary calcium intake not being different between DS and control individuals, the amount of cow's milk drunk per day was related to 25(OH)D levels, with a high percentage (18/24, 75.0%) of DS patients consuming less milk and showing 25(OH)D levels in the range of a deficiency or severe deficiency. In particular, only 30% of children with a severe deficiency (*n* = 3/10) consumed more than 200 mL of cow milk per day versus 71.4% of children (*n* = 5/7) with sufficient or insufficient 25(OH)D levels (*P* = 0.002). These data are similar to what is seen in the controls, with a high percentage (38/55, 69.1%) of individuals consuming less milk showing 25(OH)D levels in the range of a deficiency or severe deficiency.

### 3.5. Baseline Physical Activity Data

The quantitative assessment of physical activity in patients with DS and the controls showed significant differences between the two groups; the percentage of current physical activity levels was significantly lower for patients with DS than for the controls (0 hours per week group: 56% and 27%, resp.; <2 hours per week group: 41% and 43%, resp.; >2 hours per week group: 3% and 30%, resp.). However, regarding the effect of hours spent outdoors (including also physical activity) on 25(OH)D levels, there was no association between the reported average daily number of hours spent outdoors and baseline 25(OH)D levels (*P* = NS), even if patients with DS who spent more than 8 hours/week outdoors showed higher 25(OH)D levels than patients with who spent fewer than 4 hours/week outdoors (21.75 ± 6.43 versus 9.87 ± 4.35 ng/mL; *P* = 0.006).

### 3.6. Correlations of Cross-Sectional Data

Evaluating correlations among 25(OH)D and age, sex, seasons, physical activity, milk intake, PTH, BMI, height, total cholesterol, triglycerides, LDL cholesterol, systolic blood pressure, diastolic blood pressure, and autoimmune disease development, we showed that 25(OH)D levels correlated inversely with PTH (*r* = −0.42, *P* < 0.005), BMI (*r* = −0.39, *P* < 0.005), physical and outdoor activities (*r* = −0.31, *P* < 0.05), milk intake (*r* = −0.30, *P* < 0.05), total cholesterol (*r* = −0.43, *P* < 0.005), LDL cholesterol (*r* = −0.28, *P* < 0.05), systolic blood pressure (*r* = −0.34, *P* < 0.005), and autoimmune diseases (*r* = −0.56, *P* < 0.005). The multivariate linear regression analyses showed that serum 25(OH)D concentration was negatively associated with BMI (*β* = 0.29, *P* < 0.005).

### 3.7. Longitudinal Evaluation

#### 3.7.1. Effect of Vitamin D Supplementation on Vitamin D Status

After supplementation with 25(OH)D and evaluating the percentages of DS patients and controls with 25(OH)D sufficiencies, insufficiencies, and deficiencies, we show that 7 (22.6% versus 6.5%; *P* < 0.001) DS patients achieved sufficient vitamin D levels, 8 (25.8% versus 16.1%; *P* < 0.05) achieved insufficient levels, 9 (29.0% versus 45.2%; *P* < 0.001) achieved deficient levels, and 7 (22.6% versus 32.2%; *P* < 0.05) still showed a severe deficiency. The results were significantly different for the healthy controls: 26 (26.3% versus 11.1%; *P* < 0.001) reached sufficient vitamin D levels, 43 (43.4% versus 33.3%; *P* < 0.05) reached insufficient levels, 21 (21.2% versus 35.4%; *P* < 0.005) reached deficient levels, and 9 (9.1% versus 35.4%; *P* < 0.001) reached severe deficient levels.

However, in terms of 25(OH)D levels in DS subjects at the end of intervention, even if the level was significantly ameliorated (20.15 ± 10.88 versus 14.34 ± 8.31 ng/mL; *P* < 0.05), these patients still showed extremely reduced levels compared with the controls (28.27 ± 7.96; *P* < 0.0001) (Figure 1(b)). In DS patients, 25(OH)D levels do not continue to be different between males (19.35 ± 9.63 ng/mL) and females (21.08 ± 12.55; *P* = NS) and children (19.00 ± 11.49 ng/mL) and adolescents (22.09 ± 9.72; *P* = NS), whereas we showed significant statistical differences between DS subjects characterized by a normal weight (23.50 ± 11.06 ng/mL) and individuals who were obese (14.80 ± 8.86; *P* < 0.05) (Figure 2(b)) and DS individuals with (12.07 ± 6.81 ng/mL) and without (28.41 ± 8.19 ng/mL; *P* < 0.001) a history of autoimmune diseases (Figure 3(b)).

#### 3.7.2. Effect of Follow-Up on Dietary Calcium Intake

Regarding dietary calcium intake, even if calcium intake was not significantly different between DS patients and controls (846 ± 256 versus 889 ± 221 mg/day), we showed an increased but nonsignificant change in calcium intake with respect to baseline values. However, the percentage of patients and controls who drank more than 200 mL of cow's milk per day was significantly increased: in DS patients, 16/31 (51.6%) individuals continued to consume fewer than 200 mL of cow's milk for day, a better outcome than the 75% of DS patients reported in the first evaluation (*P* < 0.0001). This aspect was similar in the controls, with a significant amelioration of the percentage of people consuming more than 200 mL of cow's milk per day (27.2% versus 38.4%; *P* < 0.05). Nevertheless, milk consumption per day was always related to 25(OH)D levels, with a high percentage (14/16, 87.5%) of DS patients consuming less milk showing 25(OH)D levels in the range of a deficiency or severe deficiency.

#### 3.7.3. Effect of Follow-Up on Physical Activity

The quantitative assessment of physical activity in patients with DS and controls confirmed a significantly lower percentage of physical activity in patients with DS than the controls (0 hours per week group: 49% and 28%, resp.; <2 hours per week group: 43% and 48%, resp.; >2 hours per week group: 8% and 24%, resp.). However, regarding the effect of the number of hours spent outdoors (including also physical activity) on 25(OH)D levels, there was no association between the reported average daily hours spent outdoors and baseline 25(OH)D levels (*P* = NS), even if patients with DS who spent more than 8 hours/week outdoors showed higher 25(OH)D levels than patients with a history spending fewer than 4 hours/week outdoors (26.58 ± 9.00 versus 15.64 ± 11.71 ng/mL; *P* < 0.005).

#### 3.7.4. Effect of Vitamin D Supplementation on Bone Metabolism

DS patients still had significantly higher PTH levels compared with controls (43.57 ± 14.05 versus 26.89 ± 13.56 pg/mL; *P* < 0.005) (Figure 5(b)). However, DS subjects who were obese still showed significantly higher PTH levels (54.90 ± 13.45 pg/mL) than DS individuals with normal weights (36.50 ± 9.04 pg/mL; *P* < 0.005). However, DS patients with a history of autoimmune diseases showed higher PTH levels (56.70 ± 11.98 pg/mL) than patients without a history of autoimmune diseases (35.37 ± 7.51 pg/mL; *P* < 0.05).

#### 3.7.5. Correlations of Longitudinal Data

In evaluating the correlations among 25(OH)D and age, sex, seasons, physical activity, milk intake, PTH, BMI, height, total cholesterol, triglycerides, LDL cholesterol, systolic blood pressure, diastolic blood pressure, and a history of autoimmune disease, we showed that 25(OH)D levels were still inversely correlated with PTH (*r* = −0.38, *P* < 0.05), BMI (*r* = −0.43, *P* < 0.005), physical and outdoor activity (*r* = −0.34, *P* < 0.05), total cholesterol (*r* = −0.33, *P* < 0.05), LDL cholesterol (*r* = −0.27, *P* < 0.05), systolic blood pressure (*r* = −0.30, *P* < 0.05), milk intake (*r* = −0.36, *P* < 0.05), and autoimmune diseases (*r* = −0.59, *P* < 0.005).

## 4. Conclusions

Our study shows, for the first time, an extensive evaluation of 25(OH)D status in children and adolescents with DS. We demonstrate a very high prevalence of vitamin D deficiency in different age groups, revealing an important health problem in these patients.

In the control subjects, different seasons influenced vitamin D status [42, 43]. As with general population, for DS individuals, the 25(OH)D values differed according to the seasons, even if these values remain always less than in the control population, demonstrating the role of many different determinants and/or more determinants that more severely affected vitamin D status in these patients.

Possible reasons for this very high and important prevalence of hypovitaminosis D in these subjects, such as seen in the general population [44], may include increased urbanization, an increased time spent indoors, and extensive use of sunscreens but also a lower intake of calcium and vitamin D.

Our data confirm that DS subjects commonly spent less time outdoors and less time being physically active, important contributors to being overweight and/or obese, all factors contributing to reduced 25(OH)D values. Our data also show that DS subjects who are obese with a history of autoimmune diseases showed very reduced 25(OH)D levels, conditions very frequently seen in these patients [45].

Consistent with other authors [46, 47], we also demonstrate an inverse association between milk intake and a 25(OH)D deficit, although our result is of limited statistical significance due to the small-number statistics of our study.

As shown in recent studies in obese children without DS, we also show that obese DS children and adolescents were at a higher risk of a more severe vitamin D deficiency. The explanation for this deficiency, shared in common with the general population, stems from the decreased vitamin D bioavailability from cutaneous and dietary sources because of its deposition in body fat and because obese children may lead a more sedentary, indoor lifestyle [48, 49].

In a longitudinal study conducted with 12 DS subjects, Zubillaga et al. demonstrated that the supplementation of 800 I.U. of 25(OH)D plus 1 g of calcium once daily may yield an improvement in the biochemical markers related to the phosphocalcium metabolism and bone remodelling [26]. However, del Arco et al., studying 21 patients with DS, found no child with DS exhibiting values below the normal range, either in vitamin D metabolites or in the other parameters of calcium metabolism. Interestingly, the authors also found that the normal increment of 25(OH)D values from March to October was not observed in five children. We do not know if these subjects were obese or had a history of autoimmune diseases [27].

In DS individuals, our data show that vitamin D supplementation did not appear to be sufficient, even if 25(OH)D levels increased significantly after supplementation. However, patients with DS who were also obese and/or had a history of autoimmune diseases seem to need more 25(OH)D supplementation. These data confirm the need to extend vitamin D prophylaxis in all DS children, particularly for the high-risk population of obese individuals and subjects with autoimmune diseases. In this group of patients, we suggest using a higher dose of 25(OH)D than 400 I.U. Finally, our data showed that a 25(OH)D deficiency was associated with elevated PTH hormone levels, thus confirming the importance of a sufficient vitamin D status to maintain a normal bone metabolism. Furthermore, we found that our data showed a correlation between 25(OH)D deficiency and other cardiovascular risk factors (systolic blood pressure and LDL cholesterol level).

In fact, vitamin D deficiency has been added as a novel risk factor for cardiovascular disease [50–52], possibly by the downregulation of many genes, including those involved in renin production, proliferation of cardiac and vascular muscle cells, downregulation of C reactive protein and other proinflammatory markers [50]. Vitamin D deficiency has also been reported to be associated with a higher risk of metabolic syndrome and hypertension [50, 53].

However, epidemiological and observational studies have kindled a growing interest in the potential role of vitamin D and inflammatory process in the pathogenesis, prevention, and control of many autoimmune diseases, such as type I DM [54], multiple sclerosis, Crohn's disease, or rheumatoid arthritis. Epidemiological evidence suggests that adults with high blood levels have the lowest risk of developing multiple sclerosis or rheumatoid arthritis. However, animal and human studies seem to suggest that vitamin D is a potential modifier of diabetes [55–57], showing the possible immunomodulatory and anti-inflammatory effects of vitamin D in the reduction of autoimmune insulitis of type I DM [34, 35]. Moreover, children who show signs of vitamin D deficiency have a 2.4-fold increased risk of developing type I DM [57].

Therefore, in DS patients, reduced levels of 25(OH)D may predispose individuals to developing autoimmune diseases. However, it is also interesting to note that the presence of autoimmune disorders may increase this defect, causing other health problems in these subjects.

Finally, 25(OH)D and PTH are important to determine normal bone modeling and remodeling and insure normal bone accrual and muscle-skeletal function [58]. In DS individuals, muscle hypotonia, low levels of physical activity, poor calcium and vitamin D intake, hypogonadism, growth retardation, and thyroid dysfunction may all contribute to substantial impairments in skeletal maturation and bone-mass accrual, potentially predisposing these patients to fragility and fractures [2]. However, it is interesting to note that more and more data in recent years have showed that DS is surely a genetic form associated with an impaired bone status, such as demonstrated by densitometric data evaluated by DXA [4–7], pQCT [8], and QUS [9], although many studies have focused on adults who live either in the community or in residential institutions [10]. In fact, in the murine DS model Ts65Dn, the low BMD was correlated with significantly decreased osteoblast and osteoclast development, decreased bone biochemical markers, and a diminished bone formation rate [59]. In these mice, BMD was significantly increased after 4 weeks of intermittent PTH treatment [59]. Recently, low BMD in adults with DS has been discovered to be correlated with a significant decrease in bone formation markers, compared to controls without DS, suggesting a diminished osteoblastic bone formation and inadequate accrual of bone mass [60].

In conclusion, our results indicate that hypovitaminosis D is very frequent in DS individuals and that it is critical to assess the importance of vitamin D prophylaxis in these subjects, in particular individuals who are obese and have a history of autoimmune diseases. The reduced 25(OH)D levels seem to be also related to reduced dietary intake and outdoor activity levels. DS patients who are obese and who have a history of autoimmune diseases may need more 25(OH)D supplementation.

## Figures and Tables

**Table 1:** Biochemical and demographic parameters in Down syndrome and healthy controls at baseline and at the end of the intervention.

	Baseline		End of the study	
	Down	Controls	Down	Controls
25(OH)D, ng/mL	14.34 ± 8.31	27.04 ± 7.47^***^	20.15 ± 10.88°	28.27 ± 7.96^###^
Children	14.26 ± 8.71	27.89 ± 6.00^***^	19.00 ± 11.49	28.99 ± 6.22^###!!!^
Adolescents	14.45 ± 8.15	20.34 ± 8.45^∗∗∧∧^	22.09 ± 9.72°	23.55 ± 9.03
Total calcium, mmol/L	2.42 ± 0.14	2.51 ± 0.10	2.44 ± 0.31	2.54 ± 0.19
Phosphorous, mmol/L	1.32 ± 0.24	1.30 ± 0.32	1.31 ± 0.33	1.32 ± 0.39
Bone specific alkaline phosphate, U/L	58.3 ± 20.1	100.1 ± 31.2^***^	78.1 ± 25.6°	100.9 ± 35.7^##^
Parathyroid hormone, pmol/L	54.76 ± 32.15	26.13 ± 10.76^***^	43.57 ± 14.05	26.89 ± 13.56^###^
Cholecalciferol dietary intake, IU/day	130 ± 38	143 ± 46	164 ± 46°°	179 ± 63^§§§^
Calcium intake, mg/day	796 ± 283	821 ± 256	846 ± 256	889 ± 221
Children	810 ± 270	835 ± 285	864 ± 231	898 ± 200
Adolescents	755 ± 290	790 ± 235	812 ± 277	856 ± 231
Systolic BP, mmHg	115.9 ± 8.7	111.3 ± 8.4^*^	113.1 ± 7.7	111.6 ± 7.5
Diastolic BP, mmHg	68.2 ± 8.9	65.7 ± 8.3	66.7 ± 7.9	65.1 ± 8.0
Total cholesterol, mmol/L	4.05 ± 0.57	3.43 ± 0.61^***^	3.73 ± 0.58°	3.27 ± 0.52^§^
LDL cholesterol, mmol/L	3.00 ± 0.50	2.82 ± 0.48	2.85 ± 0.56	2.78 ± 0.60
Triglycerides, mmol/L	1.68 ± 0.48	1.53 ± 0.41	1.51 ± 0.39	1.60 ± 0.37

Down syndrome versus controls cross-sectional evaluation: ^**^ *P* < 0.005; ^***^ *P* < 0.0005.  ^∧^Controls (children) versus controls (adolescents) cross-sectional evaluation: ^∧∧^ *P* < 0.005. °Down syndrome versus Down syndrome longitudinal evaluation: °*P* < 0.05; °°*P* < 0.005. ^#^Down syndrome versus controls longitudinal evaluation: ^##^ *P* < 0.005; ^###^ *P* < 0.0005. ^§^Controls versus controls longitudinal evaluation: ^§^ *P* < 0.05;  ^§§§^ *P* < 0.0005. ^!^Controls (children) versus controls (adolescents) longitudinal evaluation: ^!!!^ *P* < 0.0005.
